# Zone 2/3 lesion and emergency repair as potential mortality predictors of TEVAR for thoracic aortic pseudoaneurysm

**DOI:** 10.1186/s13019-023-02345-8

**Published:** 2023-10-25

**Authors:** Hui Wang, Chang Shu, Tun Wang, Hao He, Xin Li, Quanming Li, Yuan Peng, Lunchang Wang, Likun Sun

**Affiliations:** 1 Department of Vascular Surgery, The Second Xiangya Hospital of Central South University, Changsha, 410011 Hunan China; 2 Vascular Diseases Institute of Central South University, Changsha, Hunan China; 3 Department of Vascular Surgery, National Center for Cardiovascular Disease, Fuwai Hospital, Chinese Academy of Medical Sciences and Peking Union Medical College, Beijing, 100037 China

**Keywords:** Aortic pseudoaneurysm, Aortoesophageal fistula, Thoracic endovascular aortic repair

## Abstract

**Objective:**

Thoracic aortic pseudoaneurysm (TAP) is an uncommon but life-threatening condition. The present study aimed to investigate the early and midterm clinical outcome of TAP patients following TEVAR and identify potential mortality predictors.

**Methods:**

We retrospectively reviewed a series of 37 eligible patients with TAP admitted to our hospital from July 2010 to July 2020. We explored their baseline, perioperative and follow-up data. Fisher exact test and Kaplan–Meier method were applied for comparing difference between groups.

**Results:**

There were 29 men and 12 women, with the mean age as 59.5 ± 13.0 years (range 30–82). The mean follow-up period was 30.7 ± 28.3 months (range 1–89). For early outcome (≤ 30 days), mortality happened in 3 (8.1%) zone 3 TAP patients versus 0 in zone 4 (*p* = 0.028); postoperative acute arterial embolism of lower extremity and type II endoleak respectively occurred in 1(2.7%) case. For midterm outcome, survival at 3 months, 1 year and 5 years was 88.8%, 75.9% and 68.3%, which showed significant difference between zone 2/3 versus zone 4 group (56.3% vs. 72.9%, *p* = 0.013) and emergent versus elective TEVAR group (0.0% versus 80.1%, *p* = 0.049). Previous stent grafting or esophageal foreign body with Aortoesophageal fistula (AEF), and systemic vasculitis, as etiologies, resulted in encouraging immediate outcome but worse midterm prognosis.

**Conclusion:**

TAP lesions at zone 2/3 and emergent TEVAR predict worse midterm outcomes compared to zone 4 lesions and elective TEVAR. The outcomes are also mainly restricted by the etiology of the TAP.

## Introduction

Considering the diversity of the natural history of TAP resulting from various etiologies [1–8], to date there is no consensus on the management of it. At present, available treatment options for TAP located at distal aortic arch and descending aorta mainly include optimal medical treatment, open surgery, and endovascular therapy. Gandhi et al. found no statistically significant difference between optimal medical treatment and thoracic endovascular aortic repair (TEVAR) in traumatic TAP patients, but its primary end points were in-hospital mortality and complications without longer follow-up [9]. The American Association for the surgery of trauma (AAST) trails [10] reported higher mortality and paraplegia rates for blunt aortic injury following open management than TEVAR, while recent research has shown favorable early and late outcomes of open repair for acute blunt aortic injury, with 5- and 10-year survival as 86–98% and 68–92% [11].

Thoracic endovascular treatment, as an alternative to surgical intervention, is increasingly more common for aortic pathologies owing to its less invasive nature. However, little evidence has been published about the early and midterm follow-up results of TAP treated by TEVAR. More literatures are needed to prove its security and efficacy [12, 13]. The paper retrospectively presents 37 patients diagnosed with TAP following TEVAR therapy. We analyzed their clinical features, early and midterm results as well as potential mortality predictors.

## Materials and methods

This retrospective study is approved by the institutional research ethics committee of our hospital and waiver of informed consent form of individuals are granted.

### Patients

**Fig. 1:**
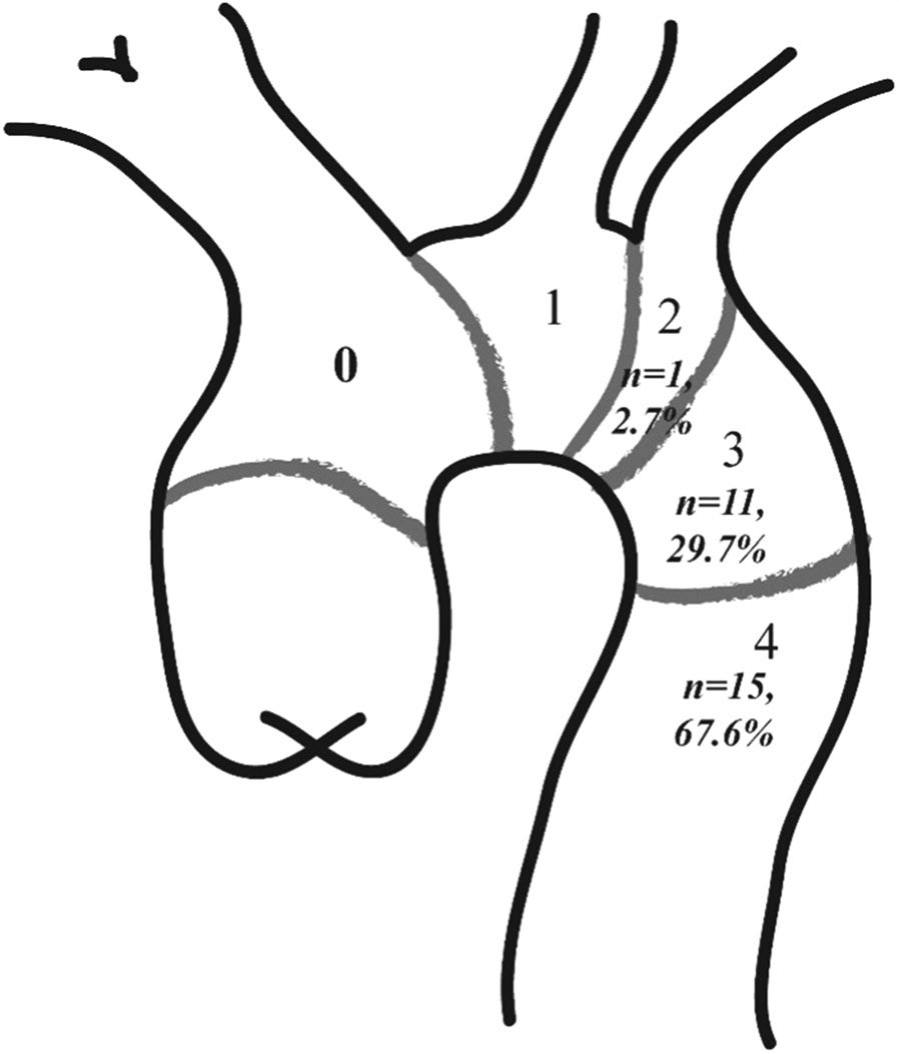
Locations of TAP lesions according to Ishimaru classification

We included patients if (1) they were diagnosed with TAP through contrast-enhanced computerized tomographic angiography (CTA), and digital substraction angiography (DSA) result was also necessary when following endovascular therapy; (2) they received TEVAR for TAP. We excluded patients if thoracic aortic lesion was induced by malignant etiologies. From July 2010 to July 2020, 37 eligible patients were selected into the study. Mean age was 59.5 ± 13.0 years (range 30–82). There were 29 males (78.4%) and 18 smokers (48.6%). The etiology was atherosclerosis in 13 (35.1%), blunt trauma in 7 (18.9%), tuberculosis in 7 (18.9%), esophageal foreign body in 4 (10.8%), systematic vasculitis in 3 (8.1%), previous stent grafting in 2 (5.4%) and unknown cause in 1 (2.7%). Based on Ishimaru classification [14], TAP lesions located at zone 2 in 1 (2.7%), zone 3 in 11 (29.7%), zone 4 in 15 (67.6%) (Fig. 1). 31 patients (83.8%) had obvious symptom while 6 patients (16.2%) were asymptomatic. More detailed information is listed in Table 1.

**Table 1:** Baseline characteristics of patients (n = 37)

Varibles	Values or n (%)
Age (years)	59.5±13.0 (30–82)
Male	29 (78.4)
Smoking	18 (48.6)
Etiology	
Atherosclerosis	13 (35.1)
Trauma	7 (18.9)
Tuberculosis	7 (18.9)
Esophageal foreign bodies	4 (10.8)
Systemic vasculitis	3 (8.1)
Previous stent grafting	2 (5.4)
Unknown	1 (2.7)
Location of pseudoaneurysm	
Zone 2	1 (2.7)
Zone 3	11 (29.7)
Zone 4	25 (67.6)
Clinical manifestation	
Chest/back pain or tightness	21 (56.8)
Haematemesis, hematochezia or melena	8 (21.6)
Trachyphonia	4 (10.8)
Hemoptysis	2 (5.4)
Fever	2 (5.4)
Cough and Sputum	1 (2.7)
Asymptomatic	6 (16.2)
Comorbidities	
Hypertension	18 (48.6)
Coronary artery disease	4 (10.8)
Diabetes	3 (8.1)
Cardiac valvular disease	1 (2.7)
Cardiac arrythmia	1 (2.7)
Renal insufficiency	1 (2.7)

### Thoracic endovascular aortic repair

Treatment with antihypertensive drugs and beta blockers to strictly maintain the systolic pressure at about 100 mm Hg and the heart rate at 60 to 70 beats/min was initiated on admission. Procedures were performed in the hybrid unit under general or local anesthesia. The right common femoral artery (CFA) was exposed through incision, approximately 5 cm, and then guidewire and catheters were inserted into CFA using puncture technique until reached appropriate sites. The left CFA was available when necessitating bilateral intervention. Digital subtraction angiography (DSA) of the thoracic aorta was performed to compare the lesion’s dynamic characteristics with those seen on CTA. After intravenous administration of heparin sodium (0.5 ml/kg), the stent graft with the diameter 10–20% oversized was advanced and prepositioned into the proper location. DSA was performed again to evaluate configuration of stent, endoleak and patency of branch vessels. Adjunctive procedures of TEVAR include physician-modified fenestration (PMF) and perfusing drugs into aneurysm sac.

PMF technique was selected to preserve LSA when the distance from TAP to LSA was < 15 mm, the neck length of the TAP was < 15 mm and the diameter was > 5.5 cm or rapid aortic growth had occurred (> 1 cm/year). The detailed process of PMF technique was same as before [15]. Generally, the outer sheath of the stent was moved 3–4 cm backward to expose the stent, a scalpel and scissor were used to remove the member of pre-fenestration area, and then using a belt to constrain the stent to return the outer sheath to the original position. Adjust the stent-graft to fit LSA orifice under radioscopy. Balloon expandable stent for LSA was deployed when necessary. On the other hand, LSA was directly covered when lack of enough landing zone if the patient had dominant right vertebral artery and no history of left internal mammary artery graft or the need of left arm access dialysis.

For patients meeting the diagnostic criteria for infection, adjunctive procedure is the inserting of a 5F sheath into the contralateral groin to enable the catheter to be preplaced in the aneurysmal sac for perfusion of anti-TB drugs or sensitive antibiotics after deploying the stent graft as reported before [16]. Patients with localized lesions and disappeared active bleeding into the lesions after stent implantation are the ideal candidates for this adjunctive procedure to ensure the seal and stay of antibiotics in the pseudoaneurysm sac. TEVAR was performed under local anesthesia when perfusing drugs to the sac was needed, for early recognition of relevant intraoperative complications. Additionally, in our experience, for traumatic patients with pleural effusion, perioperative antibiotic was necessary for preventing postoperative infection.

### Follow-up

Patients were scheduled for CTA and physical examination 1 months, 3 months, 6 months, 12 months after discharge, and annually afterwards to evaluate general condition, patency, and position of vascular graft as well as early and late complications. Telephone interview was conducted semiannually.

### Statistics

Measurement data are expressed as the mean ± standard deviation and range; enumeration data are expressed as number and proportion. Fisher exact test and Kaplan–Meier method were applied for comparing difference between subgroups. Differences in survival between the groups were analyzed using the log-rank test and breslow test. Statistics analysis was performed with SPSS 18.0 (SPSS Inc, Chicago, IL) and R software (R Core Team, Vienna, Austria).

## Results

### Early outcomes

**Fig. 2:**
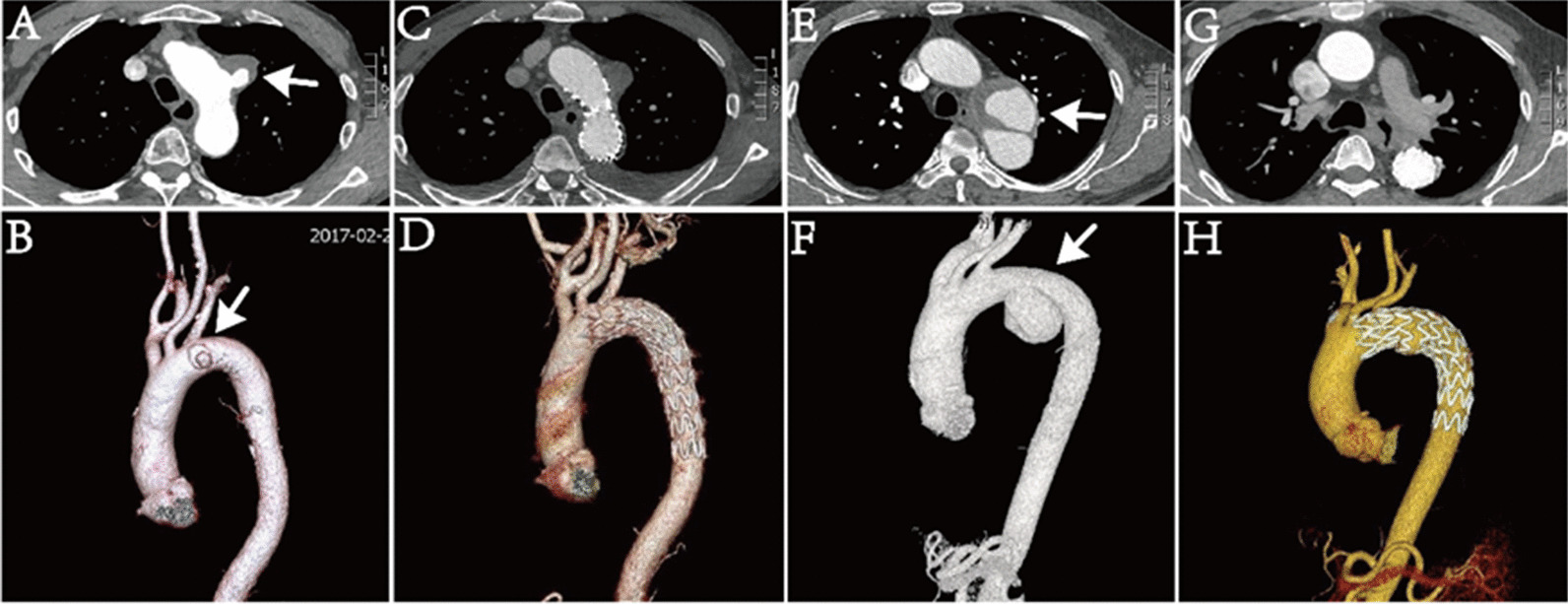
**A**–**D** Preoperative and 1-week postoperative CTA images of a thoracic TAP patient receiving TEVAR and in vitro fenestration of LSA. **E**–**H** Preoperative and 5-year postoperative CTA images of a thoracic TAP patient undergoing TEVAR

TEVAR procedure was successfully performed in 37 patients including 7 (18.9%) emergent and 30 (81.1%) elective TEVAR. LSA was covered in 11 cases (29.7%). PMF for LSA was applied in one patient (2.7%, Fig. 2A–D).

Early mortality (≤ 30 days) happened in three zone 3 TAP patients versus 0 in zone 4 (*p* = 0.028), and 2 of them received emergent TEVAR (Table 2). One case died of acute arterial embolism of the lower extremity 7 days after TEVAR, whose preoperative CTA showed extensive aortic calcification; one case with preoperative hydropericardium, hydrothorax and compressed main pulmonary artery died of pericardial tamponade and compressed main pulmonary artery the day after TEVAR, whose TAP etiology was atherosclerosis. The death of third case happened after discharge who had stable physical condition when discharged. For the complications, Type II endoleak was observed in one zone 4 TEVAR case, which was left untreated and expected to be thrombosed spontaneously. For overall patients having received TEVAR within 30 days, there was no occurrence of ischemia of left upper extremity, stroke, paralysis, acute renal insufficiency, bowel ischemia or puncture complications, and the 30-days survival was 88.8% (Table 2).

**Table 2:** Surgery related details and clinical outcome in zone2/3 and zone4 APA patients (n (%))

Variable	Zone2/3 APA (n = 12)	Zone4 APA (n = 25)	Total (%) (n = 37)	*p*
Surgery opportunity				0.406
Emergent TEVAR	3^a^	4	7 (18.9)	
Elective TEVAR	9^b^	21	30 (81.1)	
Stent type				
Ankura, Lifetech	5	14	19 (51.4)	
Valiant, Medtronic	3	6	9 (24.3)	
Hercules, Microport	3	3	6 (16.2)	
Zenith TX2, Cook	1	1	2 (5.4)	
Relay, Bolton	0	1	1 (2.7)	
Early mortality	3	0	3 (8.1)	0.028
Early morbidity				0.642
Acute arterial embolism of lower extremity	1	0	1 (2.7)	
Type II endoleak	0	1	1 (2.7)	
Secondary intervention	0	1^c^	1 (2.7)	0.676
Midterm survival				0.013^d^
3 months	65.6 ± 14.0	100.0 ± 0.0	88.8 ± 5.3	
6 months	56.3 ± 14.8	100.0 ± 0.0	85.8 ± 5.9	
1 year	56.3 ± 14.8	85.0 ± 8.0	75.9 ± 7.5	
3 years	56.3 ± 14.8	85.0 ± 8.0	75.9 ± 7.5	
5 years	56.3 ± 14.8	72.9 ± 13.2	68.3 ± 9.9	

^a^Postoperative in-hospital death happened in one case

^b^One zone 2 case received PMF to preserve LSA

^c^Exploratory thoracotomy and aortoesophageal repair 5 months after TEVAR

^d^Kaplan-Meier method with Breslow test

### Midterm outcomes

**Fig. 3:**
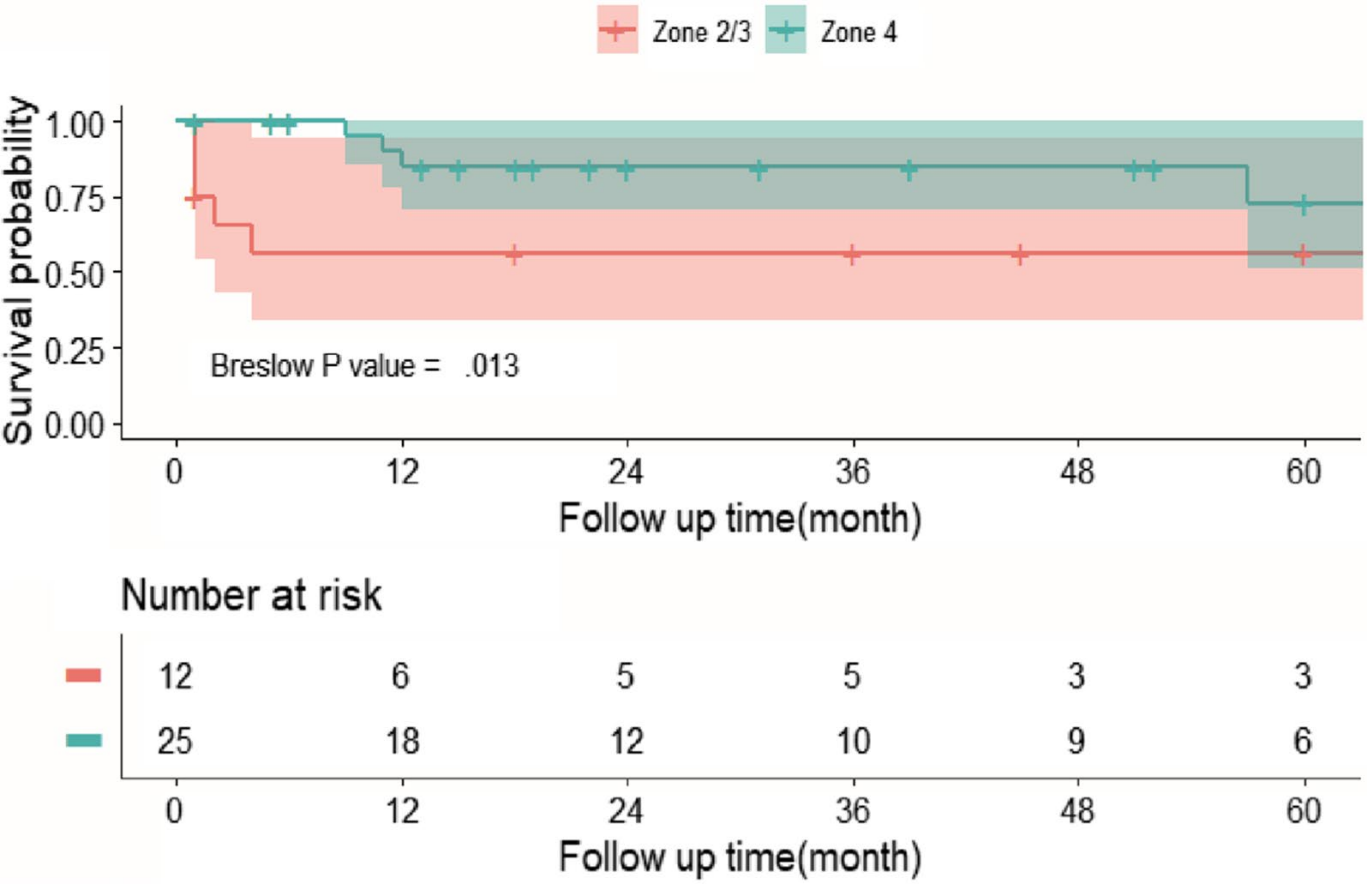
Kaplan–Meier survival curve based on subgroups with TAP lesions located at different Ishimaru zone

The mean follow up was 30.7 ± 28.3 months (range 1–89), and the overall survival at 3 months, 6 months, 1 year, 3 years and 5 years was 88.8%, 85.8%, 75.9%, 75.9% and 68.3%. No death occurred in zone 4 group within 6 months, and the overall survival showed significant difference between zone 2/3 and zone 4 groups (*p* = 0.013) (Table2, Fig. 3). Six new adverse events happened during midterm follow up, 4 of which had known cause. One case developed aortoesophageal fistula (AEF) due to esophageal foreign body, manifested as “hematochezia for 2 days, 79 g/l hemoglobin”. He received emergent TEVAR and strict perioperative anti-infection treatment, but subsequently, he suffered from recurrent fever, was treated by exploratory thoracotomy and aortoesophageal repair 5 months later, but finally died of sepsis because of stent infection after 57 months. The other case was also diagnosed with AEF caused by previous stent grafting due to aortic dissection three years ago. His massive hematemesis needed emergent TEVAR, but he died of bacteremia 11 months after TEVAR. The third one developed TAP due to connective tissue disease, who died of aortic rupture 12 months after TEVAR. The last case had satisfactory follow-up results but died of other disease 9 months later. There was no complication or reintervention in other patients. For special case, the patient following PMF remains good condition after 45-months follow up. Representative midterm postoperative (5 years) CTA results of a trauma patient was showed in Fig. 2E–H.

### Analysis of mortality predictors

**Fig. 4:**
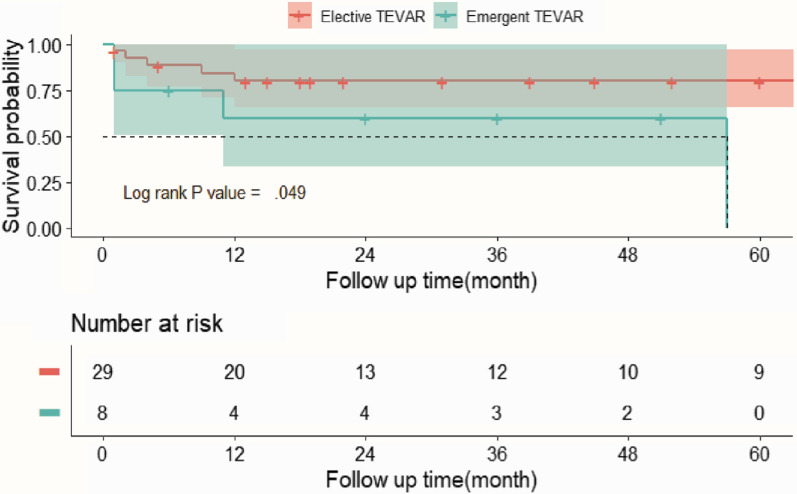
Kaplan–Meier survival curve based on subgroups with elective TEVAR versus emergent TEVAR for TAP patients

According to Kaplan–Meier analysis with study endpoint as 60 months, locations of thoracic TAP lesions and surgery opportunity had significant different effects on patients’ midterm survival outcome (Fig. 3, 4).

Patients with zone 4 TAP had more favorable survival than zone 2/3 TAP, especially in the early stage (breslow test, *p* = 0.013) (Fig. 3). The specific survival for zone 4 patients at 6 months, 1 year, 3 years and 5 years was 100.0%, 85.8%, 85.8% and 72.9%, while that for zone 2/3 patients at 3 months and 6 months, and 5 years was 65.6%, 56.3% and 56.3% (Table 2). The adverse events of zone 4 group happened to 2 connective tissue disease caused TAP patients who respectively died of aortic rupture and other disease as mentioned above; also happened to 2 AEF patients with previous stent grafting and esophageal foreign body who respectively died of bacteremia and sepsis. The adverse events of zone 2/3 group happened to 4 atherosclerosis caused TAP patients and one of them died of postoperative acute arterial embolism of the lower extremity; also happened to one tuberculosis caused TAP patient.

Patients received elective TEVAR had more satisfactory survival than emergent TEVAR cases (log-rank test, *p* = 0.049) (Fig. 4). The detailed survival rate for elective TEVAR patients at 1 month, 3 months, 6 months, 1 year, 5 years was 96.6%, 92.5%, 88.5%, 80.1% and 80.1%. However, the survival for emergent TEVAR patients at 1 month, 1 year and 5 years was 75.0%, 60.0% and 0.0%, respectively. For most emergent cases, life-threatening postoperative adverse events happened within a year, and then patients’ condition got stabilized. One emergent case died after 57-months follow up due to sepsis who had received TEVAR because of esophageal foreign body cause, as mentioned above.

**Fig. 5:**
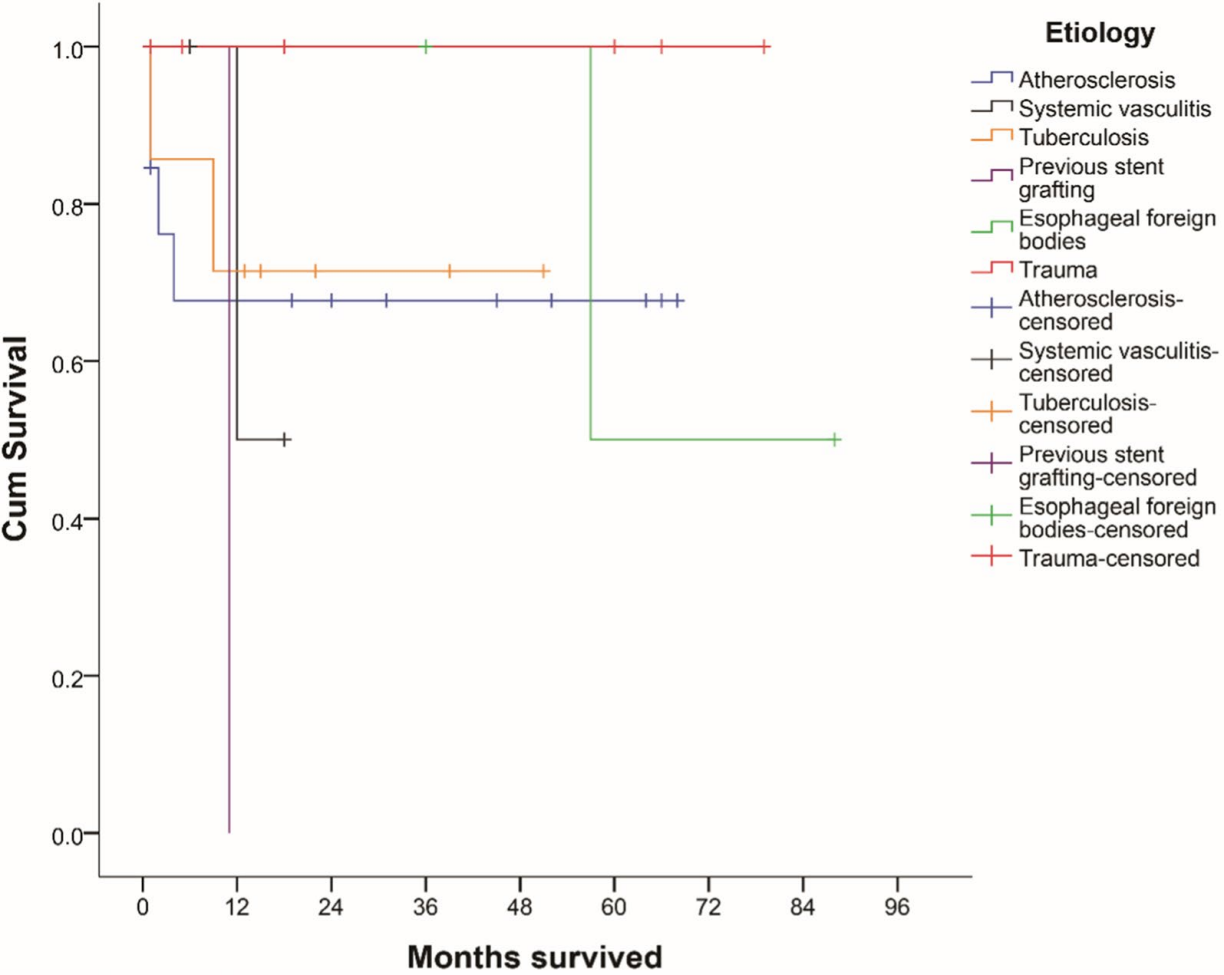
Kaplan–Meier survival curve based on subgroups with different etiologies of TAP patients

According to the survival curve regarding the etiologies of TAP, patients with post-traumatic TAP had the best midterm survival: no death or complication was observed during 79-months follow up (Fig. 5). The survival of 13 TAP patients with atherosclerosis cause at 1 month and 68 months was 84.6% and 67.7%. The survival of TAP patients with tuberculosis cause at 1 month and 51 months was 85.7% and 71.4%. The midterm survival of TAP patients caused by previous stent grafting, esophageal foreign bodies and systemic vasculitis was unsatisfactory, respectively as 0.0%, 50.0% and 50.0%. In our series, AEF is shared and concomitant anatomical change for previous stent graft implantation and esophageal foreign bodies caused TAP patients.

As the multivariate Cox regression results, certain factors posed increased risk to worse survival outcome including zone2/3 lesion (HR 4.605, 95% CI 1.095–19.359), emergent TEVAR (HR 4.196, 95% CI 1.042–16.891) and concomitant cardiac disease (HR 4.932, 95% CI 1.086–22.403) (Table 3).

**Table 3:**
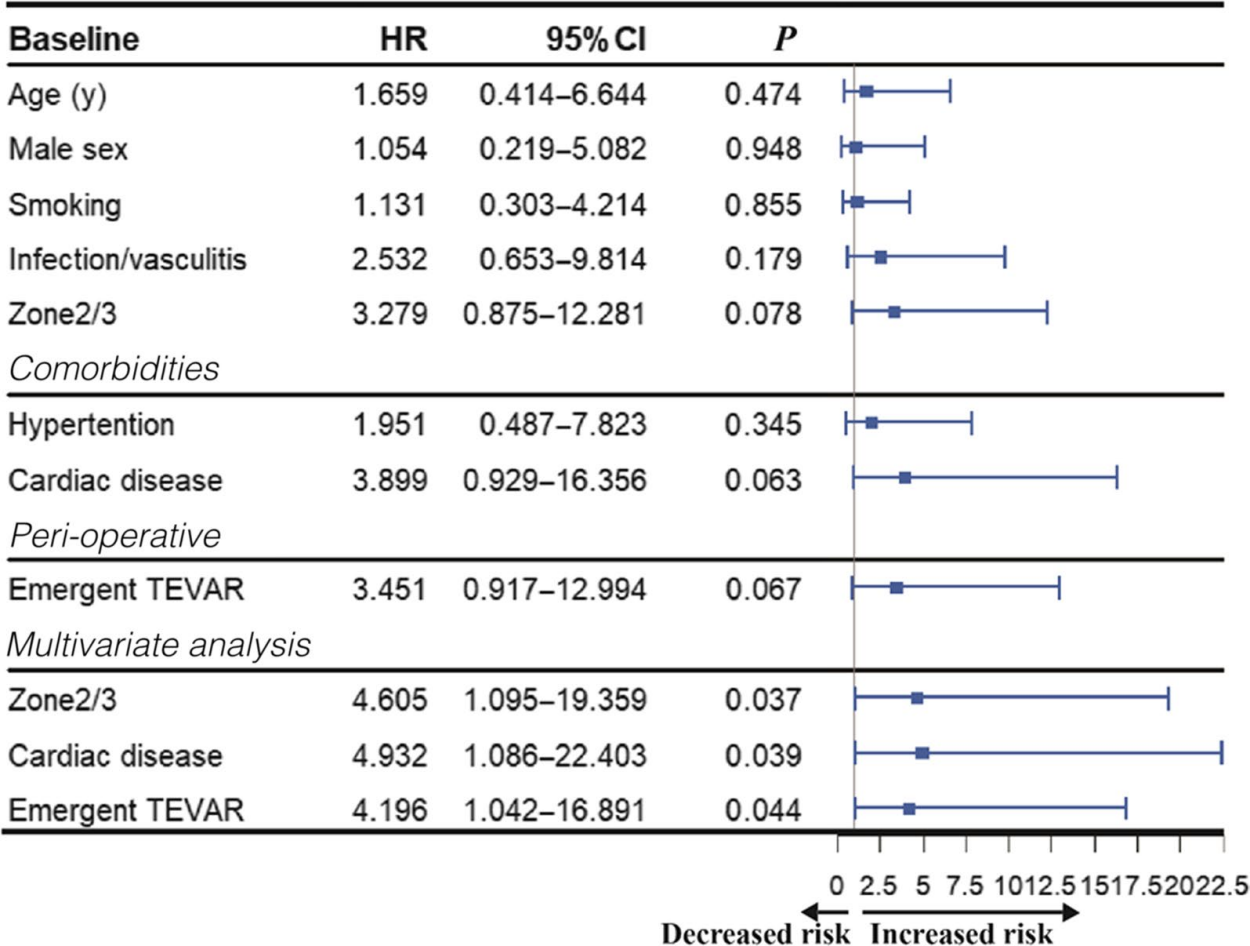
Risk fator analysis of survival outcome for thoracic APA patients after TEVAR

## Discussion

In this paper, we described early and midterm results of TAP patients following TEVAR therapy. As a procedure with less invasiveness, short procedure duration and less bleeding, TEVAR is an ideal alternative of open surgery for aortic pathologies at distal aortic arch and descending aorta, especially for emergent situation with combined traumatic injury, severe infection, and aortic rupture. Therefore, TEVAR should be considered for TAP considering its emergency and complexity. The present series showed that the 5-years survival of TAP patients following TEVAR was 68.3%, and the outcomes are mainly restricted by the etiologies. To some extent, lesions at zone 2/3 and emergent TEVAR predict worse midterm outcomes compared to zone 4 lesions and elective TEVAR.

In this series of TAP, the survival at 1 and 5 years was 75.9% and 68.3%, which was a satisfactory result comparing to open surgery [17]. The mortality of over 10% at 3 months might links to patients’ complex condition, emergent surgical treatment and early complication of TEVAR. As research showed that once emergent cases survived TEVAR procedure, the survival of them become stable after 6 months [18]. The Kaplan–Meier curve demonstrated that great survival discrepancy exists between TAP patients with different etiologies signifying characteristic pathogenesis (Fig. 5).

Atherosclerotic thoracic TAP accounts for the largest proportion (13, 35.1%) in our series. Actually, atherosclerotic aortic pseudoaneurysm generally originates from penetrating aortic ulcer (PAU) [2]. We usually make the judgment based on patients’ advanced age, the gravity of aortic calcification by CTA and medical history of aortic ulcer or intramural hematoma by CTA results. A study showed that the survival of atherosclerotic TAP after TEVAR has a gradual yearly decreasing trend with 5-years survival lower than 40% due to extensive atherosclerosis of the aortic wall [18]. The trend is unobvious in our results with 68-months survival as 67.7%, which means that patients with TAP caused by atherosclerosis might be the ideal candidates for TEVAR therapy. In addition, one atherosclerotic TAP case in our series with extensive aortic calcification died of acute embolism of lower extremity. Therefore, like well-studied periprocedural stroke due to the displacement and embolization of aortic debris after TEVAR [19], perioperative embolism of lower extremity also deserves more investigation.

Blunt trauma is a mostly reported pathogenesis, and it has been a trend to use TEVAR to treat trauma related aortic pathologies. 7 (18.9%) trauma TAP cases in our series all received TEVAR therapy, and no complication or death was observed during the follow up (Fig. 5). The result is understandable due to healthier and younger vessel nature based on trauma patients’ relative younger ages (52.9 ± 9.6 years, 44–68) than atherosclerotic patients (66.9 ± 8.1 years, 56–82). According to the literatures, the line between nonoperative management (NOM) and operative treatment for grade III (pseudoaneurysm) blunt traumatic aortic injury (BTAI) remains blurred. Some studies suggested that BTAI with pseudoaneurysm could be managed by NOM instead of instant operative treatment [9, 20]. However, there was 10% risk of TAP requiring surgical therapy after NOM [20], and postdischarge follow-up was restricted to < 1 year in most studies [21]. Based on the outcomes of our series, we suggest that instant TEVAR for traumatic TAP is reasonable.

Infected TAP is a tough condition with high mortality [22–25]. These microorganisms reach the vessel wall through various ways including direct invasion and spread, feeding vessels, lymphatic vessels, and iatrogenic factors. Despite the controversy of treatment with artificial graft exposed to infected tissue, many studies have shown that endovascular repair is a palliative treatment in acute phase, a temporary bridge to secondary open surgery [22, 26–28]. For patients only undergoing endovascular treatment, life-long appropriate antibiotic therapy, or regular and full-course anti-tuberculous chemotherapy is recommended [29]. In our center, innovative adjunctive procedure of TEVAR, as mentioned in the part of methods and recently published article [30], was adopted for eligible patients with infection cause or high risk of postoperative infection cause, like esophageal foreign body. After the deployment of aortic stent sealing the aneurysm sac, directly delivering the antibiotic agent to the aneurysmal sac via pre-placed catheter would ensure that the drugs would permeate into nearby tissues persistently and slowly instead of being swept away by blood stream. Moreover, in situ administration improve drug bioavailability compared to oral or intravenous administration.

In our series, none of the patients was the direct consequence of non-tuberculous bacterial infection, while tuberculosis TAP patients account for 18.9% (7 cases). Satisfactory early and midterm outcome had been obtained: patients gained weight during follow up, with 1 month and 51 months survival as 85.7% and 71.4%. Different from usual acute bacterial infection, infection with mycobacterium tuberculosis is a chronic reaction generating no endotoxin, exotoxin, invasive enzyme, flagellum and spores, with lipid as its chief pathogenic substance. Additionally, novel delivery system of anti-tuberculosis drugs to improve their bioavailability is a research focus currently [31], while in situ administration reduces isonicotinic acid hydrazide and rifampicin acid interaction in the stomach so as to avoid inadequate RIF bioavailability and drug resistant tuberculosis [32]. The above factors might explain why tuberculous TAP patients are ideal candidates for TEVAR with adjunctive procedure. Furthermore, despite that the intraluminal thrombus help prolong duration of drug action, the adjunctive procedure could also cooperate with state-of-the-art drug delivery system to further prolong the action time.

Systemic vasculitis is another intractable pathogenesis of TAP, and the outcome of them was unsatisfactory albeit following immunotherapy (Fig. 5). Some experience on TEVAR for systemic vasculitis has been reported, which is proved superior to open surgery on preventing immediate complications and aortic anatomical pseudoaneurysm. However, recurrent TAP remains to be a continuing problem because of aortic wall injury or mechanical force at the edge of the stent triggering vascular inflammation [33]. It is suggested that adjunctive perioperative and postoperative immunosuppressive treatment is essential [34, 35].

TAP patients caused by previous TEVAR and esophageal foreign body developed concomitant AEF. Theoretically, TAP could be the cause or independent of AEF. On one hand, TAP can result from stent graft that causes tight adherence and pressure necrosis of the esophageal wall, and then the mechanical compression and secondary invasion from TAP lead to AEF, which takes a relatively longer time. On the other hand, TAP can result from local infection accompanied with AEF, which takes shorter time. For patients with previous TEVAR, TAP is considered as the cause of AEF considering that the time to the observation of TAP after first TEVAR respectively was 39 months and 8 years, and one patient had 2-years dysphagia history. For patients with esophageal foreign body, TAP might be a simultaneous course with AEF considering that non-sterile foreign body can cause local infection. TEVAR has limited effect on both situations due to the inability to eradicate the underlying etiology. One patient with previous TEVAR died of bacteriemia 11 months after secondary TEVAR. Postoperative recurrent systemic infection happened in one patient with esophageal foreign body who died of sepsis 57 months after TEVAR. Although studies showed that aortic fistula caused by ingestion of a foreign body are the ideal candidates for TEVAR with encouraging immediate outcomes, caution must be stressed for the postoperative infectious complications [36, 37]. AEF patients following open management also had poor outcome including implantation of vascular prosthesis and patch repair [38]. The latest research indicated that in situ aortic replacement by cryopreserved aortic homograft and concomitant primary closure of the esophagus is a feasible and promising therapy for primary and secondary AEF [39].

Our results suggest that patients with zone 4 TAP have a significantly more favorable midterm outcome than zone 3 TAP. A similar study also indicated the existence of better death/survival ratio among patients with middle thoracic aneurysms than among patients with aneurysms situated at proximal aorta [40]. For one thing, when the orifice of LSA that was only partially covered by the stent, local fluid dynamics showed remarkable disturbance [41]. For another thing, zone 4 aneurysms, with sufficient proximal landing zone, could receive a straightforward endovascular surgery without advanced adjunctive procedures and thus avoid relevant complications, having greater chance of satisfactory survival [40].

Symptomatic cases accounts for 83.8% (31/37) of overall patients. The most common symptom was chest/back pain or tightness (56.8%, 21/37). It was back pain and distress in another series [18]. Other symptoms include hemoptysis, hematemesis, hematochezia and melena, which are strikingly noticeable as quite common symptoms of respiratory and digestive diseases. In our series, TAP with massive hemoptysis, haematemesis or hematochezia was indications for emergent TEVAR, besides ruptured descending thoracic aortic aneurysms, acute BTAI and complicated acute type B aortic dissections [42]. Massive hemoptysis, haematemesis or hematochezia occurred due to aortopulmonary fistula (APF) or AEF even with a visible intrabronchial or intraesophageal mass [26, 43–48]. There was no detectable APF in our patients manifested as hemoptysis, albeit in one patient who complained of persistent hemoptysis for even seven years after TEVAR. Under this circumstances, respiratory disorder is more likely to be considered.

Although there are several results in this report, there are also limitations. It is a retrospective observational study based on single center experience with a long-time span and limited cases. Despite that the patient series displayed a wide spectrum seen in clinical practice, the result might lack adequate statistical power to decide important clinical differences confidently. Definitive conclusions on the effectiveness and mortality predictors of TEVAR for TAP need further studies with larger patient populations and longer follow-up.

## Conclusion

TAP lesions at zone 2/3 and emergent TEVAR predict worse midterm outcomes compared to zone 4 lesions and elective TEVAR. The outcomes are also mainly restricted by the etiology of the TAP.

## Data Availability

The data in this article come from the patient’s dataset of the Second Xiangya Hospital. Please contact the authors for the original data.
